# Ecological divergence of burying beetles into the forest canopy

**DOI:** 10.7717/peerj.5829

**Published:** 2018-11-15

**Authors:** Jillian D. Wettlaufer, Kevin W. Burke, Adam Schizkoske, David V. Beresford, Paul R. Martin

**Affiliations:** 1 Department of Biology, Queen’s University, Kingston, ON, Canada; 2 Department of Biology, Trent University, Peterborough, ON, Canada

**Keywords:** *Nicrophorus*, Burying beetle, Competition, Resource partitioning, Entomology, Carrion beetle, Silphidae, Community ecology, Species coexistence, Species interactions

## Abstract

Closely related species with overlapping geographic ranges encounter a significant challenge: they share many ecological traits and preferences but must partition resources to coexist. In Ontario, potentially eleven species of carrion beetles (Coleoptera: Silphidae) live together and require vertebrate carrion for reproduction. Their reliance on an ephemeral and uncommon resource that is unpredictable in space and time is thought to create intense intra- and interspecific competition. Evidence suggests that burying beetle species reduce competition by partitioning carrion for breeding across different habitats, temperatures, and seasons. Here, we test predictions of an alternative axis for partitioning carrion: vertical partitioning between the ground and forest canopy. We conducted a survey of carrion beetles from May to July 2016 at the Queen’s University Biological Station across 50 randomly generated points using baited lethal traps at zero and six metres. Ground traps yielded more species and individuals compared to those in the canopy, and the number of individuals and species caught increased through the season in both trap types. Ground and canopy traps were accurately distinguished by the presence or absence of three species: ground traps contained more *Nicrophorus orbicollis* and *Necrophila americana*, while canopy traps contained more *Nicrophorus pustulatus*. We trapped 253 *N. pustulatus* in the canopy, but only 60 on the ground. *N. pustulatus* is thought to be rare across its geographic range, but our results suggest it is uniquely common in canopy habitats, demonstrating a vertical partitioning of habitat and resources. Our results are consistent with *N. pustulatus* having diverged into canopy habitats as a strategy to coexist with closely related sympatric species when competing for similar resources. We still, however, do not know the traits that allow *N. pustulatus* to flourish in the canopy, exactly how *N. pustulatus* uses canopy resources for breeding, or the factors that restrict the expansion of other burying beetles into this habitat.

## Introduction

The coexistence of closely related species presents an ecological challenge: they share many traits and preferences through recent common ancestry and often depend on similar resources, and yet are thought to require ecological partitioning to allow them to coexist (Weber & Strauss, 2016). For this reason, burying beetles in the family Silphidae have been a focus of ecological studies of resource partitioning—they all require vertebrate carcasses for reproduction and these carcasses are thought to be limiting resources in their environment (Wilson, Knollenberg & Fudge, 1984; Anderson & Peck, 1985; Trumbo & Bloch, 2002). Despite these similar requirements, many different Silphid species co-occur within communities, sometimes fighting over the same resources (Wilson, Knollenberg & Fudge, 1984; Otronen, 1988; Trumbo & Bloch, 2002). Do these species partition carrion to coexist within communities, and, if so, how? Previous studies have suggested an answer to this question: burying beetles may coexist because they partition carrion based on habitat, timing of seasonal activity and breeding, timing of diel activity, and trade-offs between the ability to locate carcasses quickly vs competitive dominance (Anderson, 1982; Wilson, Knollenberg & Fudge, 1984; Trumbo, 1990; Trumbo & Bloch, 2002; Beninger & Peck, 1992; Lingafelter, 1995; Trumbo & Bloch, 2000, 2002; Urbanski & Baraniak, 2015). These axes of ecological partitioning are plausible hypotheses to explain how different burying beetle species coexist, but the ecological distinctions of some species remain poorly known.

The ecology of one species in particular, *Nicrophorus pustulatus*, remains enigmatic. Historically, *N. pustulatus* was found to be widespread but rare across its range because few individuals were caught in ground traps typically set for burying beetles (Anderson & Peck, 1985). However, the regular occurrence of *N. pustulatus* at lights (Anderson, 1982; Lingafelter, 1995) was difficult to explain. A later discovery of *N. pustulatus* feeding on gray ratsnake (*Pantherophis spiloides*) eggs (Blouin-Demers & Weatherhead, 2000; Keller & Heske, 2001) led some researchers to suggest that *N. pustulatus* specialized on ratsnake eggs, or oviparous snake eggs in general; this idea was supported by an observation of *N. pustulatus* larvae feeding on northern ringneck snake (*Diadophis punctas edwardsii*) eggs (LeGros, Pratt & Beresford, 2010) and an unpublished report of them feeding on eastern fox snake (*Pantherophis gloydi*) eggs (Brown & Beresford, 2016). However, the range of *N. pustulatus* extends beyond that of oviparous snakes (Anderson & Peck, 1985), and its occurrence in a failed Northern Saw-whet Owl (*Aegolius acadicus*) nest eight metres high in the forest canopy in Connecticut, USA was inconsistent with *N. pustulatus* specializing on snake eggs (Philips, Root & DeSimone, 1983). The use of vertebrate carrion by *N. pustulatus* in captivity (Robertson, 1992; Trumbo, 1992, 2007; Rauter & Moore, 2002; Rauter & Rust, 2012) also suggested that this species might use vertebrate carrion in nature similar to other *Nicrophorus* species.

Further studies have reported *N. pustulatus* to be more common in the forest subcanopy and canopy, rather than on the ground where most *Nicrophorus* species breed and feed. *N. pustulatus* was first caught in the canopy in general insect traps (e.g., flight intercept traps; Ulyshen & Hanula, 2007). Following this work, burying beetle surveys using baited traps found *N. pustulatus* almost exclusively several meters above the ground (Ulyshen, Hanula & Horn, 2007; LeGros & Beresford, 2010). These canopy *N. pustulatus* would have gone undetected using ground-based surveys, such as carrion baited pit-fall traps (Su & Woods, 2001; Schroeder, Buddle & Saint-Germain, 2009), that were typically used because burying beetles were thought to require ground soil for burying and reproduction. To date, five studies have used baited traps to sample burying beetles in the canopy, and all have detected *N. pustulatus*. Ulyshen, Hanula & Horn (2007) discovered *N. pustulatus* was more abundant in canopy traps, where 33 individuals were found at five and 15 m, and only one individual was caught at 0.5 m above the ground. LeGros & Beresford (2010) found *N. pustulatus* also preferred canopy habitats, where six individuals were caught in six m traps and none at two and four m. Lowe & Lauff (2012) investigated arboreal carrion use by suspending baited nest boxes 9–10 m in the canopy and found *N. pustulatus* on six occasions (with no observations of reproductive behavior), representing fewer than 4% of beetle encounters with carrion in their study. Dyer & Price (2013) collected 106 individuals at 2.5 m compared to 47 individuals at 0.5 m in surveys in Maryland, USA. Brown & Beresford (2016) collected perhaps the greatest number of *N. pustulatus*; they captured 174 individuals in six m traps, representing 13.3% of their total *Nicrophorus* catch, in Thousands Islands National Park, Ontario and Brockville, Ontario. Overall, *N. pustulatus* has been repeatedly caught in elevated traps, but only rarely in large numbers.

Here, we test the hypothesis that *Nicrophorus pustulatus* is primarily found in the canopy, and that the burying beetle community differs in species occurrence and abundance between the ground and the canopy. To test these hypotheses, we employed paired, baited traps on the ground and six metres off the ground at our study site in southeastern Ontario, Canada. Because burying beetles typically breed on the ground (Anderson & Peck, 1985), we predicted that (1) traps on the ground would yield more species and individuals compared to those in the canopy, (2) ground and canopy traps would differ in their composition and abundance of species, and (3) canopy traps would collect a greater abundance of *Nicrophorus pustulatus* compared to ground traps.

## Methods

### Study species

The carrion beetle family, Silphidae, is comprised of two subfamilies: Silphinae and Nicrophorinae. Species in the subfamily Silphinae may avoid competition with Nicrophorinae species by using larger carcasses, whereas *Nicrophorus* prefer smaller carcasses that can be buried more easily (Anderson & Peck, 1985). Nicrophorinae, also known as the burying beetles, exhibit unique resource guarding and parental care behavior. Burying beetles breed on small vertebrate carcasses and typically avoid competition with flies and other scavengers by burying or covering the carcass (Anderson & Peck, 1985). If more than one pair of adult beetles is present on the carcass, including pairs of different species, fighting typically ensues and continues until only one pair remains (Anderson & Peck, 1985). The winning pair then rears and cares for their offspring, using the carcass to feed their larvae. In southeastern Ontario, potentially 11 species of carrion beetles (Anderson & Peck, 1985) live closely together and compete for small vertebrate carrion. These carrion beetles show evidence of both spatial and temporal habitat partitioning (for details, see Anderson, 1982; Wilson, Knollenberg & Fudge, 1984; Anderson & Peck, 1985; Beninger & Peck, 1992; Scott, 1998; Trumbo & Bloch, 2000; Sikes, Trumbo & Peck, 2016).

We included all species of carrion beetles from the family Silphidae that we caught during our study. These species included six in the genus *Nicrophorus* (Nicrophorinae): *Nicrophorus orbicollis, N. pustulatus, N. tomentosus, N. sayi, N. defodiens*, and *N. hebes*, and four species from the subfamily Silphinae: *Necrophila americana, Necrodes surinamensis*, *Oiceoptoma inaequale*, and *O. noveboracense*.

### Study site

We collected burying beetles in lethal traps baited with chicken on the Queen’s University Biology Station (QUBS, 44.5653, −76.322, 129 m) properties near Elgin, Ontario, Canada during the reproductive period from early May until late July 2016. We set traps at 50 block-randomized points across QUBS properties that are the subject of long-term studies of diverse taxonomic groups (birds, plants, and insects). These study points were originally chosen by randomly selecting GPS points that fell within the property boundaries, with the restriction that no point could fall within a body of water, and each point was at least 400 m away from all other points. Our study site includes areas of regrowth forest dominated by sugar maple (*Acer saccharum*) and ironwood (*Ostrya virginiana*), with some species of ash (*Fraximus* spp.), elm (*Ulmus* spp.), hickory (*Carya* spp.), and birch (*Betula* spp.), as well as basswood (*Tilia americana*), and areas of mixed forest with coniferous trees (*Pinus* spp., *Thuja occidentalis*, *Tsuga canadensis*) (Martin, 1994). The more mature deciduous and mixed forests reach an average height of about 24 m (Jones et al., 2001). Additional trapping locations at our study site include: areas of wet woodland composed mainly of eastern white cedar (*Thuja occidentalis*) and birch species, man-made conifer plantations, edges of small lakes and beaver ponds, forest edges, open fields that were once used for agriculture, or open rocky outcrops composed of scattered red oak (*Quercus rubra*), eastern white pine (*Pinus strobus*), red (*Juniperus virginia*) and common (*J. communis*) juniper, and a number of mosses, grasses, and lichen-covered rock (Martin, 1994).

### Trapping methods

At each trapping location, we set two concurrent traps and collected them after 7 days: a pitfall trap in the ground, and a trap of the same design suspended six m above ground. We sampled each point twice: once in May/June, and once in July. We constructed our traps using plastic buckets approximately 35 cm deep and 17 cm in diameter. We filled the buckets with six cm (depth) of saturated saline solution to kill and preserve the beetles. We covered the top of each trap with a 35 cm^2^ piece of chicken wire. We baited each trap with one chicken wing wrapped in cheesecloth, suspended from the middle of the chicken wire using steel craft wire. The bait was frozen until deployed in traps without any prior thawing or ripening and suspended above the saline preservative. We covered each trap with a 30 cm^2^ plywood board to prevent rainwater from entering. We secured each ground trap by placing large rocks from each site on top of the plywood board in an attempt to deter vertebrate scavengers from disrupting the traps. Canopy traps were hung six m high in tree branches; the exact distance between the paired ground and canopy traps varied depending on the availability of soil for ground pitfall traps and trees for canopy traps (average = 4.8 m between paired ground and canopy traps with the largest distance being <20 m). Differences in the number of successful traps between the ground and canopy were caused by a greater disturbance of ground traps (*N* = 34), likely by vertebrate scavengers stealing the bait and/or pulling the trap from the ground. Three traps were also omitted because of trap failure due to human error in deployment. A total of 34 traps, mostly in the canopy, were successfully deployed and were undisturbed by vertebrates but did not collect any carrion beetles. These traps were included in tests for differences in the number of beetles and number of species between ground and canopy traps, but were omitted from subsequent classification analyses because they provided no information on carrion beetle community composition.

### Species and sex identification

Each beetle specimen was first identified as a Silphidae by their relatively large size, possession of clavate or capitate 11-segmented antennae, prominent fore coxae, and elytra that were truncate, tricostate, or lacking costae. Once identified as a Silphidae, each specimen was identified to genus, species, and sex following Anderson & Peck (1985).

### Statistical analyses

We performed all of our statistical analyses and plotting in R (R Core Team, 2016; version 3.3.1).

### Generalized linear mixed-effects models

To test our hypotheses that the number of species and abundance of burying beetles was higher in ground vs canopy traps, we ran generalized linear mixed-effects models with the number of species and total number of burying beetles as the response variables in two different models, and ground vs canopy trap and Julian date of trap retrieval as predictor variables in a saturated model. We included trapping location (station) as a random factor in each model because sampling events at the same location on different days were not independent from each other. We omitted traps that showed any evidence of disturbance, likely by vertebrate scavengers. We transformed the number of beetles [log(number of beetles + 3)] and rounded values to integers to meet the assumptions of the models. We rescaled Julian date using the *rescale* function in the R package *arm* (version 1.9–3; Gelman et al., 2016), that subtracts the mean and divides by two standard deviations, following recommendations in Bolker et al. (2009). We first ran models with a Poisson distribution using the *glmer* function in the *lme4* package (version 1.1–12; Bates et al., 2016). We ran the full model for each response variable independently and examined its fit by plotting standardized residuals against fitted values and all predictors, testing for linearity and homogeneity in the variance of residuals for each predictor using plots and Bartlett’s tests, testing if the distribution of residuals and predictors differed from normal using Shapiro–Wilk tests, and testing for overdispersion (Zuur et al., 2009). Poisson models for the number of beetles fit well. Poisson models for the number of species, however, showed non-normal residuals, so we moved to Gaussian generalized linear mixed-effects models using the *lme* function in the *nlme* package (version 3.1–128; Pinheiro et al., 2016). We checked model fit of the Gaussian models as before (omitting tests of overdispersion) (Zuur et al., 2009).

We compared the performance of models with all combinations of predictor variables using the *dredge* command in the *MuMIn* package (version 1.15.6; Bartoń, 2016) to determine the best-performing model as assessed by Akaike information criterion values, controlling for small sample size (AICc; lowest value indicating the best-performing model). We checked the fit of our best-performing models using the same approach as our full models. We present the results of our best-performing models (lowest AICc values) in this paper.

### Random forest models

To test our hypotheses that burying beetle community composition differed between the ground and the canopy, and that *N. pustulatus* was only prevalent in the canopy, we first took a machine learning approach. Specifically, we used random forest classification models in the *randomForest* package in R (Breiman, 2001; version 4.6–12; Breiman et al., 2015). Random forest models combine many classification trees to identify which variables most accurately discriminate between groups (Cutler et al., 2007). Random forest classification is a powerful alternative to traditional parametric and semiparametric statistical methods for classification and discrimination because it makes no distributional assumptions about the data (Cutler et al., 2007), and can easily accommodate non-linear relationships that are common in nature (Friedl & Brodley, 1997). The random forest algorithm selects a random subset of the data (approximately 63%) and fits a classification tree to each subsample (Cutler et al., 2007). The accuracy of each classification tree is then assessed using the remaining (unselected or “out-of-the-bag”) portion of the data (Cutler et al., 2007). The out-of-the-bag data provide independent estimates of classification accuracy because they were not used to fit the classification tree (Cutler et al., 2007). Each classification tree uses only a small number of predictor variables at a time; we identified the optimal number of predictor variables for classification as the smallest number that yielded the lowest out-of-bag estimate of error rate. After many iterations of the model (10,000 in our case), the random forest model provided an overall best classification error rate, an error rate specific to each group, the relative importance of each predictor variable for accurate classification, and other details such as the classification error rate for each individual data point (Breiman, 2001). We chose to use random forest, rather than more recent classification approaches (Hothorn et al., 2017), because random forest provided more intuitive output and plots, and our analysis was relatively simple (bivariate classification, all continuous predictors using the same scale) and thus did not suffer from some of the limitations of the random forest approach (Strobl et al., 2007).

In our random forest models, we used trap height classification as the response (group) variable and the average number of carrion beetles of each species collected at each survey point (averaged across surveys) as the predictor variables. We omitted traps when no beetles were caught, regardless of trap disturbance, because traps with zero beetles provided no information on burying beetle community composition. We calculated the classification accuracy as 1—out-of-bag error rate and ran each model 10,000 times to obtain an average classification accuracy with 95% confidence intervals (CI). We constructed variable importance plots to show the relative importance of all predictor variables for accurate classification in our model. We also used partial dependence plots to depict the effects of our most important predictor variables on the probability of correct classification (Cutler et al., 2007).

### Binomial generalized linear models

We also tested our hypothesis that the occurrence and abundance of different beetle species predicted ground vs canopy traps using a binomial generalized linear model (i.e., logistic regression). Trap (ground = 0, canopy = 1) was the response variable, and different species’ average abundances (averaged across all surveys per site) were the predictor variables. We first ran a model with all species included separately, with no interaction terms, and checked the fit of the model using the *heatmap.fit* command in the R package *heatmapFit* (version 2.0.4; Esarey, Pierce & Du, 2016). We then compared the performance of different models with all combinations of predictor variables to identify the model that performed best (lowest AICc values) using the *dredge* command in the *MuMIn* package (version 1.15.6; Bartoń, 2016). Perfect separation in our best-performing model led to inflated and inaccurate statistical results. Thus, we used Firth’s penalized-likelihood logistic regression models to estimate coefficients and statistical results for reporting.

Our raw data and R code files are available in the Supplemental Materials.

## Results

**Figure 1:**
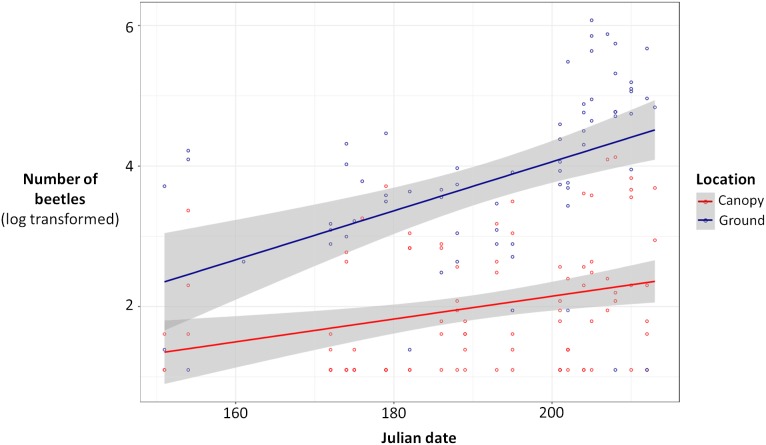
Total number of beetles [log(total number of beetles + 3)] for ground traps (blue) and canopy traps (red) by date with 95% confidence intervals (gray). The abundance of beetles was higher in ground traps compared to canopy traps (generalized linear mixed-effects model, glmm, *P* < 0.0001), and the abundance of beetles increased with Julian date for both ground and canopy traps (glmm, *P* < 0.0001).

**Figure 2:**
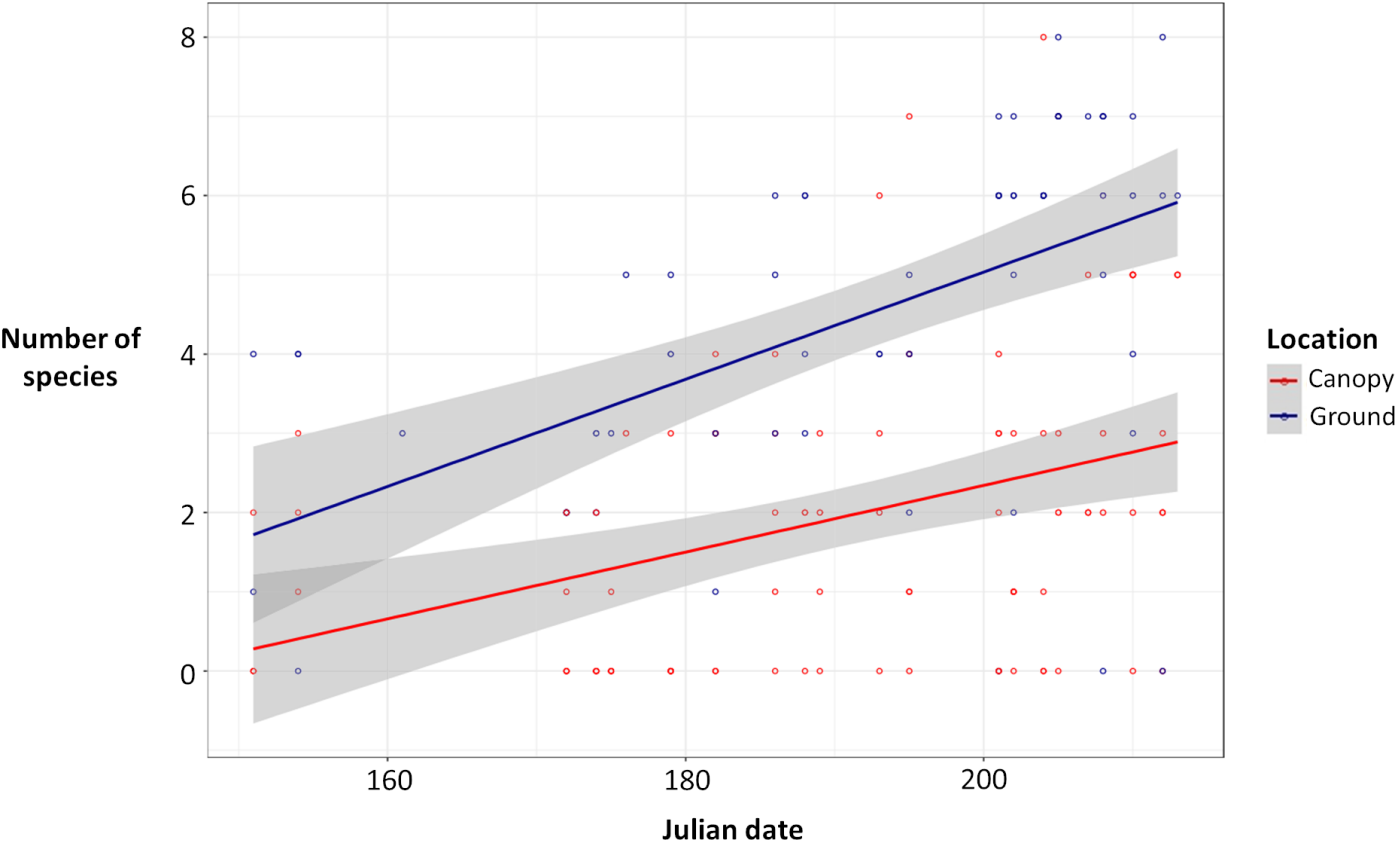
Number of species of beetles in ground traps (blue) compared to canopy traps (red) by Julian date with 95% confidence intervals (gray). Ground traps contained more species than canopy traps (glmm, *P* < 0.0001), and the number of species caught increased with Julian date for both ground and canopy traps (glmm, *P* < 0.0001).

Ground traps caught significantly more beetles compared to canopy traps (Poisson glmm, *z* = 11.49, *P* < 0.0001; Fig. 1), and the number of beetles caught in both ground and canopy traps increased with Julian date (Poisson glmm, *z* = 5.32, *P* < 0.0001; Fig. 1). Ground traps also caught significantly more species of burying beetles compared to canopy traps (Gaussian glmm, *t* = 8.82, *P* < 0.0001; Fig. 2), and the number of species in both ground and canopy traps increased with Julian date (Gaussian glmm, *t* = 6.21, *P* < 0.0001; Fig. 2).

**Figure 3:**
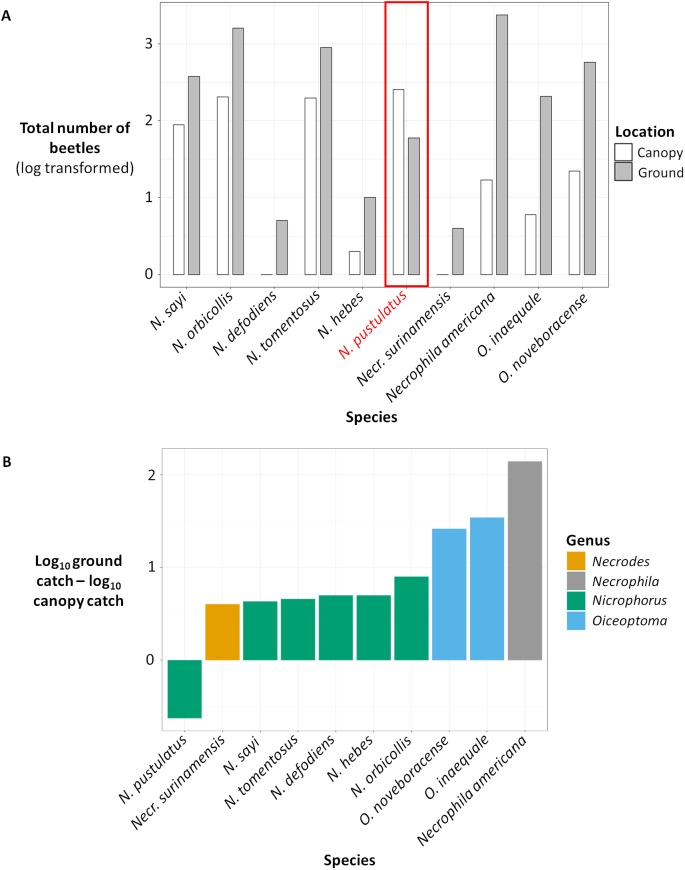
(A) Total number of beetles (log transformed) for each species in canopy traps (white) vs ground traps (gray). (B) Difference between the total ground catch and the total canopy catch (log transformed). (A) Total number of beetles (log transformed) collected for each carrion beetle species in canopy traps (white) vs ground traps (gray). (B) Difference between the total ground catch and the total canopy catch (log transformed) for each carrion beetle species, color coded by genus. The number of beetles were log transformed because some species were extremely abundant while other species were rare.

**Figure 4:**
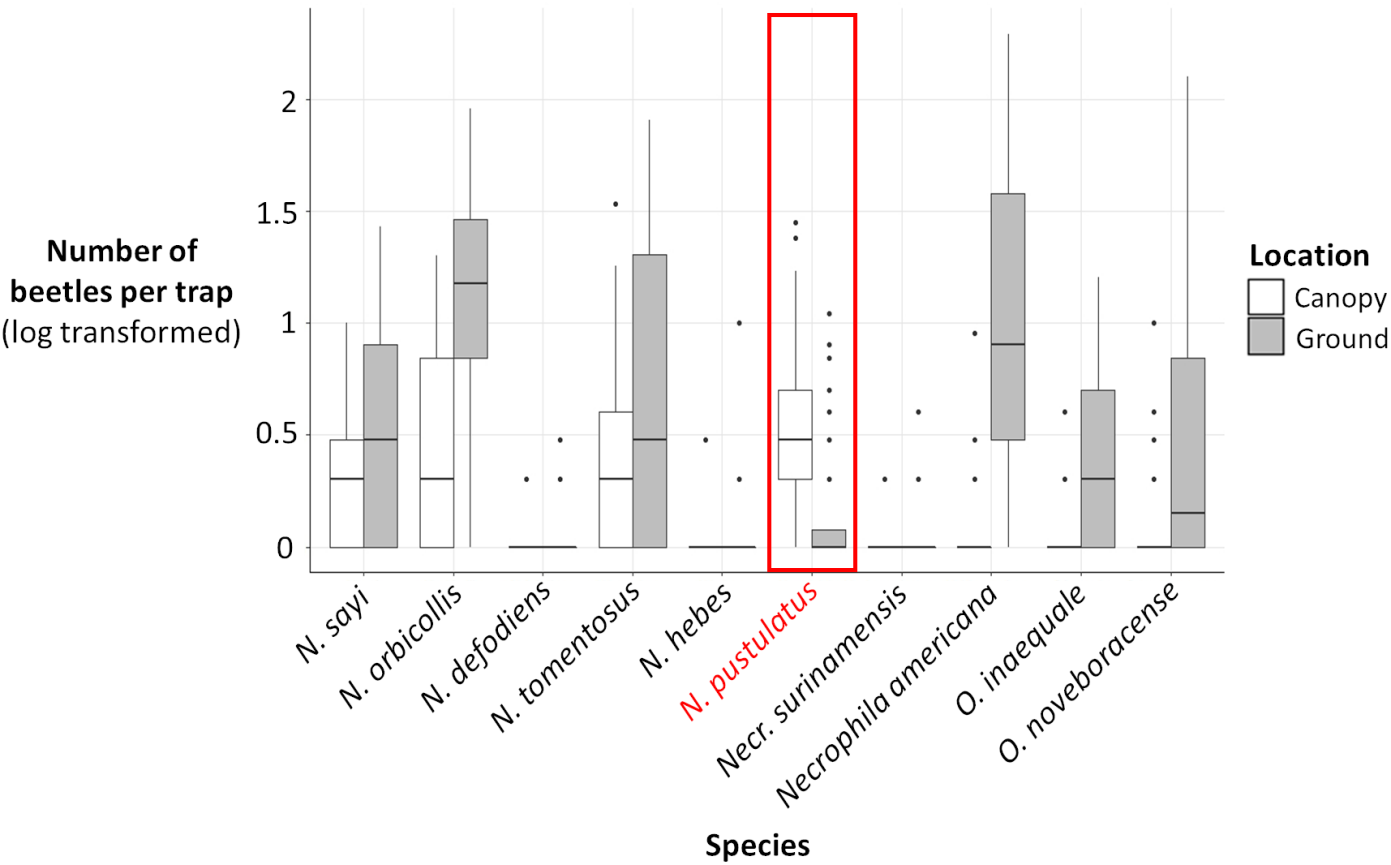
Log (number of beetles per trap + 1) for each carrion beetle species collected in canopy traps (white) vs ground traps (gray). The number of *N. pustulatus* per trap was higher in canopy traps compared to ground traps; all other burying beetle species were more common in ground traps. Boxplots show medians (thick lines), 25th and 75th percentiles (boxes), 1.5 times the interquartile range (whiskers), and outliers (points outside 1.5 times the interquartile range).

Ground and canopy traps also differed in the numbers and identities of species caught (Table 1; Figs. 3 and 4). Our most accurate random forest model correctly classified trap location as ground or canopy 92.94% of the time (95% CI [92.85–93.03]). Model accuracy was similar for ground and canopy traps; ground traps were classified correctly 91.11% of the time (CI [91.02–91.20]; *N* = 45 sites), while canopy traps were classified correctly 95.00% of the time (CI [94.95–95.05]; *N* = 40 sites).

**Table 1:** Carrion beetles captured in ground and canopy traps at the Queen’s University Biological Station (May–July 2016).

Species	Total number of beetles by trap height		Number of traps with each species present	
	Ground (0 m) *N* = 80	Canopy (6 m) *N* = 65	Ground (0 m) *N* = 80	Canopy (6 m) *N* = 65
*Nicrophorus orbicollis*	1,609	203	74 (92.5%)	40 (61.5%)
*Nicrophorus tomentosus*	902	198	47 (58.8%)	33 (50.8%)
*Nicrophorus sayi*	378	88	54 (67.5%)	34 (52.3%)
*Nicrophorus pustulatus*	60	253	20 (25.0%)	50 (76.9%)
*Nicrophorus hebes*	10	2	2 (2.5%)	1 (1.5%)
*Nicrophorus defodiens*	5	1	4 (5.0%)	1 (1.5%)
*Necrophila americana*	2,361	17	63 (78.8%)	8 (12.3%)
*Oiceoptoma noveboracense*	574	22	40 (50.0%)	9 (13.8%)
*Oiceoptoma inaequale*	207	6	47 (58.8%)	4 (6.2%)
*Necrodes surinamensis*	4	1	2 (2.5%)	1 (1.5%)
Total	6,110	791		
Total *Nicrophorus*	2,964	745		

**Note:**

*N* reflects the total number of traps that caught beetles (traps that caught no beetles were removed). In total, 100 traps were set in both the canopy and on the ground.

**Figure 5:**
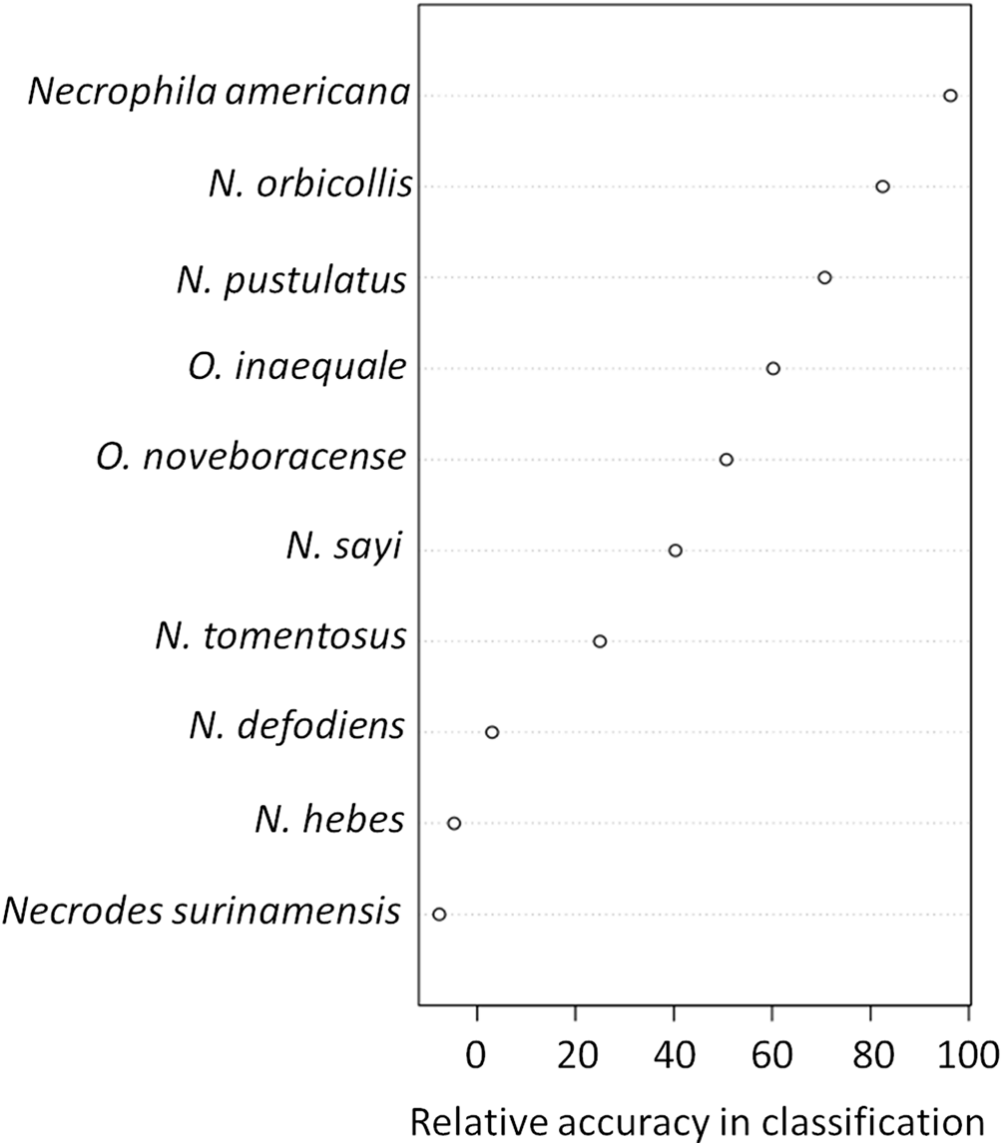
Variable importance plots for classification of ground vs canopy traps. Variables that were important for classification were those that best predicted whether a trap was on the ground or in the canopy in our random forest classification model. Specifically, the relative accuracy in classification (*x*-axis) compares the prediction error of the random forest model when applied to the portion of the dataset not used to fit the model (the “out-of-bag” portion of the data); this error is calculated as the difference between the prediction error with predictor variables intact, and the prediction error after permuting each predictor variable in the out-of-bag portion of the dataset. The difference in prediction errors are averaged across all trees and normalized (divided by the standard deviation of the differences) to create the values in the *x*-axis (Breiman et al., 2015). The best predictors of trap height were *Necrophila americana*, *Nicrophorus pustulatus*, and *Nicrophorus orbicollis*.

**Figure 6:**
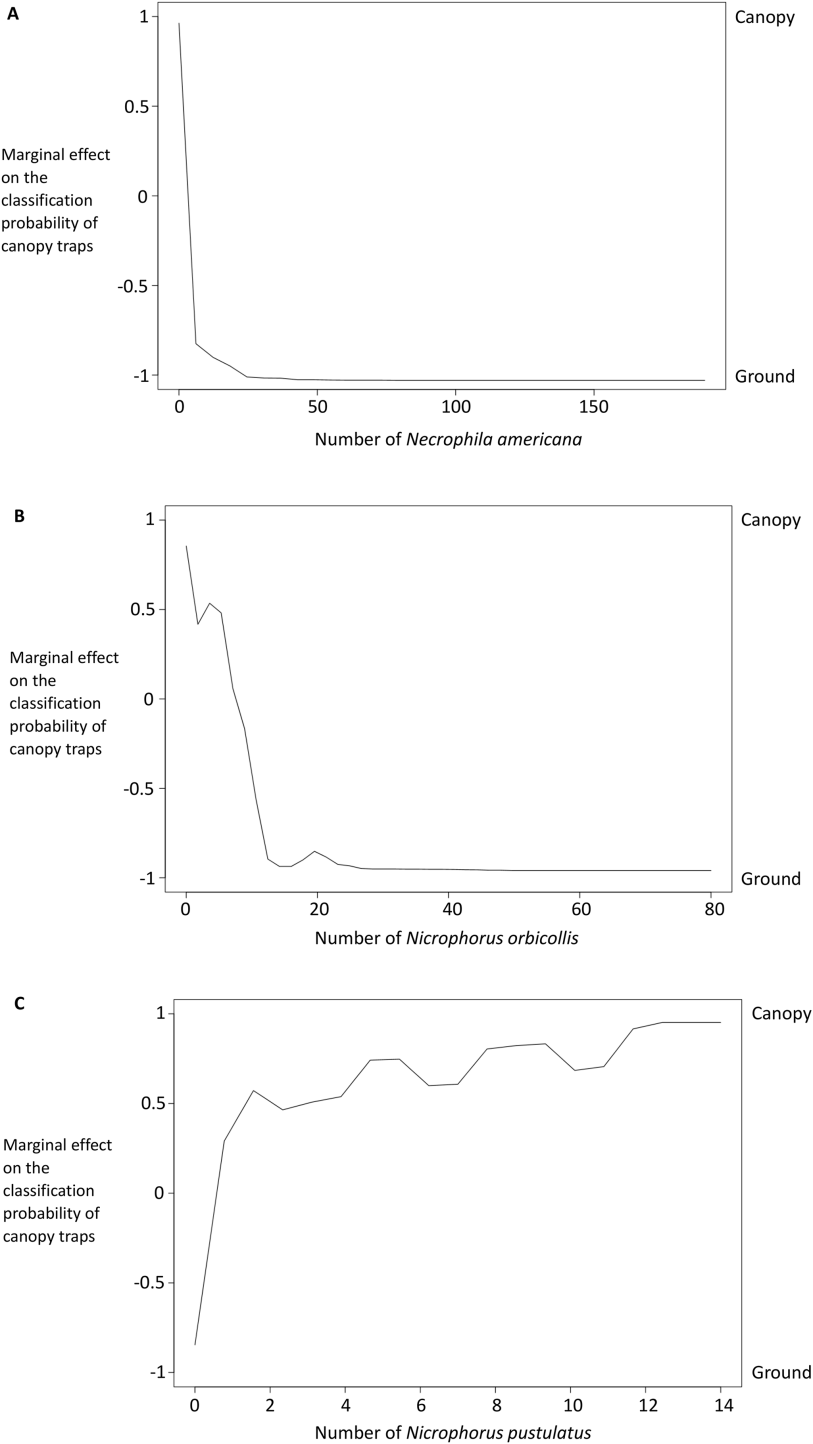
Partial dependency plots show the marginal effects of each species’ abundance on the probability that a trap was in the canopy or on the ground. The *y*-axis is the marginal effect on the classification probability of canopy traps (i.e., the relative logit contribution of the *x*-axis variable on the probability of canopy traps for different values of the *x*-axis). A negative value means that the canopy traps are less likely than ground traps for that value of the *x*-axis; a positive value means that canopy traps are more likely. See Breiman et al. (2015) for details on calculations. Traps with more *Necrophila americana* (A) and *Nicrophorus orbicollis* (B) were more likely to be ground traps; traps with more *Nicrophorus pustulatus* (C) were more likely to be canopy traps.

The presence and abundance of three species of carrion beetles were the most important predictors of trap height (Figs. 4–6). The presence of *Nicrophorus orbicollis* and, particularly, *Necrophila americana*, were the best predictors of ground traps (Fig. 5), and the likelihood of a trap being on the ground increased as abundance of both species increased (Fig. 6). The presence, and increased numbers, of *Nicrophorus pustulatus* individuals was the best predictor of canopy traps (Figs. 5 and 6). The abundance of other species also helped to accurately classify trap height, but to a lesser extent (Figs. 4 and 5), and including them reduced classification accuracy relative to some simpler models. For example, our random forest classification model rerun with only *Necrophila americana*, *Nicrophorus pustulatus*, and *Nicrophorus orbicollis* correctly classified trap location as ground or canopy 94.12% of the time, while our model rerun with only *Necrophila americana* and *Nicrophorus pustulatus* accurately classified trap location 85.88% of the time; the full model (all species included) had a classification accuracy of 92.94%.

Results from our binomial generalized linear models supported our random forest analysis. The presence and abundance of *Necrophila americana* and *Nicrophorus pustulatus* were the most statistically significant predictors of ground vs canopy traps in our best-performing model (lowest AICc; Table 2). The presence and abundance of other species of burying beetle were also significant predictors of ground vs canopy traps; however, the error associated with their effect sizes was larger (Table 2), suggesting they were less consistent predictors. The coefficient estimates for *Nicrophorus pustulatus* were very different from all other species (Table 2), illustrating that *N. pustulatus* was uniquely common in the canopy and rare on the ground (cf. Fig. 4).

**Table 2:** Results of Firth’s penalized-likelihood logistic regression testing the hypothesis that the abundance of each carrion beetle species differed between ground and canopy traps (*N* = 145 comparisons).

Firth’s penalized-likelihood logistic regression^1^					
Predictor variable	Estimate	Lower 95% CI^2^	Upper 95% CI^2^	Chi-squared	*P*
Intercept	1.21	−0.59	3.99	1.7	0.19
*Necrophila americana*	−0.98	−4.68	−0.37	24.8	<0.0001
*Nicrophorus pustulatus*	4.20	1.50	12.63	17.8	<0.0001
*Nicrophorus orbicollis*	−0.32	−1.15	0.01	3.5	0.06

**Notes:**

The model represents the best-performing logistic regression model (lowest AICc value), comparing models with all possible combinations of predictor variables.

1 implemented because of perfect separation.

2 CI, confidence interval.

## Discussion

The abundance and occurrence of burying beetle species differed between ground and canopy traps. Ground traps captured more species and more individuals of burying beetles than canopy traps. The number of individuals and species increased as the season progressed from May to June, for both ground and canopy traps, indicating that there is a greater abundance and greater species diversity later in the season. Trap height classification of ground vs canopy traps differed with species present in the trap. Our most accurate random forest model for classifying trap height was very accurate (92.94% classification accuracy), indicating there were repeatable differences between ground and canopy trap heights in the species that were caught. Ground traps were accurately predicted by the abundance of either *Necrophila americana* or *Nicrophorus orbicollis*, and greater abundances of either of these species indicated the trap was more likely to be on the ground. Canopy traps were accurately predicted by the abundance of *Nicrophorus pustulatus*; the presence and increasing abundance of *N. pustulatus* in a trap was a strong predictor of canopy traps.

Our findings support previous studies that found greater abundances of *N. pustulatus* in elevated traps (Ulyshen & Hanula, 2007; LeGros & Beresford, 2010; Dyer & Price, 2013; Brown & Beresford, 2016). *N. pustulatus* was historically thought to be rare, but widely distributed, in eastern North America (Anderson & Peck, 1985), a pattern consistent with the few individuals caught in previous studies (Anderson, 1982; Robertson, 1992; LeGros & Beresford, 2010; Brousseau, Cloutier & Hébert, 2010). Our study supports the findings of Dyer & Price (2013), suggesting that *N. pustulatus* can be common, but only within the forest canopy. Ulyshen, Hanula & Horn (2007) found 21 individuals of *N. pustulatus* in four 15 m canopy traps, and progressively fewer in the four five m traps (*N* = 12) and in the four ground traps (*N* = 1), suggesting that *N. pustulatus* may be even more abundant at greater heights above our six m canopy traps.

The only known breeding resource for *N. pustulatus* in nature is snake eggs (Blouin-Demers & Weatherhead, 2000; Keller & Heske, 2001; Smith et al., 2007; LeGros, Pratt & Beresford, 2010). However, the geographic range of *N. pustulatus* extends beyond the range of all oviparous snakes (Anderson & Peck, 1985); thus, *N. pustulatus* must use other sources of food for breeding in some parts of its range. In our study, we successfully baited *N. pustulatus* into traps using chicken, and it has been reported that in a laboratory setting, *N. pustulatus* will behave like a typical burying beetle and rear offspring on mice (Robertson, 1992; Trumbo, 1992; Rauter & Moore, 2002). Dyer & Price (2013) also trapped *N. pustulatus* using fish as bait, and suggested that *N. pustulatus* might use fish as a resource for breeding, consistent with observations of *N. investigator* (Hocking, Ring & Reimchen, 2006). Fish resources could be available in the canopy in areas of fish-eating raptor nests (Dyer & Price, 2013). Philips, Root & DeSimone (1983) discovered three adult *N. pustulatus* in a failed Northern Saw-whet Owl nest, supporting the idea that this species uses other food for breeding beyond snake eggs. In addition, a pair of *N. pustulatus* were observed and collected on dead Tree Swallow (*Tachycineta bicolor*) nestlings in a failed nest at the Queen’s University Biological Station, in a nest box approximately 1m from the ground (A. Schizkoske, 2016, unpublished data). Further studies are needed to determine the typical food used for reproduction by *N. pustulatus*.

*Nicrophorus pustulatus* may prefer canopy habitats to avoid intense competition for carrion on the ground (Ulyshen, Hanula & Horn, 2007) and to exploit important carrion resources in the canopy. Carrion in the canopy may include squirrels (Sciuridae), birds, and bats (Chiroptera) (LeGros & Beresford, 2010). In particular, nesting squirrels and birds are common in the canopy and frequently experience mortality (Ricklefs, 1969), providing a reliable resource during the peak breeding season (e.g., June at our study site for birds; Peck & James, 1987; Keast, 1990; Cadman et al., 2007). Furthermore, the breeding season of vertebrates in the canopy coincides with the emergence of *N. pustulatus* at our study site (Trumbo, 1990).

Why don’t other *Nicrophorus* species use canopy habitat given the abundance of nesting vertebrates there? *Nicrophorus* beetles typically bury carcasses under soil or leaf litter to protect them from other competitors and assist in reproduction (e.g., insulation). This burying behavior may not be possible in canopy habitats. The search for carrion in the canopy, including cavity searching, may also be more energetically costly and some *Nicrophorus* species may be unable to sustain flight for necessary periods or maneuver sufficiently to find carrion in this habitat. The use of canopy habitats in *Nicrophorus*, however, may extend beyond *N. pustulatus*. Outside of *N. pustulatus*’ range, Okawara (1991) found that *N. investigator* and *N. tenuipes* were more common in traps set at 9 and 12.6 m than those set at 0, 1.8, and 5.6 m, suggesting that vertical height partitioning may occur in Japanese (Hokkaido) carrion beetle communities as well. While we still do not understand the constraints on using canopy habitat, the use of canopy resources by *N. pustulatus*, and both *N. investigator* and *N. tenuipes* in Japan, suggests that vertical height is another important axis of resource partitioning among closely related species of burying beetles.

## Conclusions

Most species of carrion beetles in our study, and elsewhere, typically use carrion resources located on the ground. *N. pustulatus* is an exception, primarily using carrion located in canopy habitats and only secondarily using carrion on the ground. Our findings illustrate a distinct vertical axis of resource partitioning in our carrion beetle community that may allow *N. pustulatus* to co-occur with other closely related species that all require the same limited resource for reproduction.

## Supplemental Information

10.7717/peerj.5829/supp-1 Supplemental Information 1 Dataset used in our study, excluding zeros (traps that did not catch any beetles).

10.7717/peerj.5829/supp-2 Supplemental Information 2 Dataset used in our study, including zeros (traps that did not catch any beetles).

10.7717/peerj.5829/supp-3 Supplemental Information 3 R code: Test for differences between ground and canopy traps in number of species and number of beetles, random forest models, binomial GLM models.

10.7717/peerj.5829/supp-4 Supplemental Information 4 Readme file: description of variables in the datasets.

## Additional Information and Declarations

### Data Availability

The following information was supplied regarding data availability: The raw data and code are available in the Supplemental Files.
